# Bi‐Stable Metamaterials with Intrinsic Memory for Selective Wave Filtering Based on Frequency and Amplitude

**DOI:** 10.1002/advs.202405146

**Published:** 2024-11-06

**Authors:** Nathan N. Stenseng, Mahmoud M. Samak, Osama R. Bilal

**Affiliations:** ^1^ School of Mechanical, Aerospace, and Manufacturing Engineering University of Connecticut Storrs CT 06269 USA

**Keywords:** acoustic metamaterials, bistabllity, harmonic waves, programmable materials, solitons

## Abstract

Analytical, numerical, and experimental methods are used to investigate the utility of metamaterials in controlling harmonic waves based on both their amplitude and frequency. By programming the metamaterials to support bi‐stable configurations (i.e., two stable phases), the required conditions are elucidated for a transition wave (i.e., a topological soliton) to nucleate due to harmonic excitation, causing a phase change within our metamaterial. As each of these phases has its own unique transmission frequency range, such phase change is harnessed to control harmonic waves based on both their amplitude and frequency. As a demonstration of principle, a low/high‐pass filter is shown by tuning the same metamaterial to change phase; from transmission to attenuation and vice versa. In addition, phase transitions taking place while preserving the metamaterial's state of attenuation or transmission are shown. Such materials can continue their functionality (i.e., either attenuation or transmission of waves) while keeping a record of extreme events that can cause their transition (i.e., have memory). These metamaterials can be useful in the next generations of advanced and functional acoustic devices.

## Graphical abstract

By inducing phase changes in metamaterials using topological solitons, the authors show effective control of harmonic waves based on frequency, amplitude, and specific combinations of both. In addition, the metamaterial retains a record (i.e., have memory) of extreme events without changing its main functionality of either blocking or transmitting waves. The proposed platform can be utilized in the next generation of advanced acoustic devices.

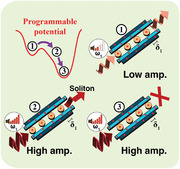

## 1 Introduction

Acoustic metamaterials are artificially engineered structures specifically designed to manipulate acoustic waves.^[^ ^1^, ^2^, ^3^ ^]^ Different unit cell geometries can produce a variety of extraordinary properties such as tunable reciprocity,^[^ ^4^, ^5^, ^6^, ^7^, ^8^ ^]^ bi‐stability,^[^ ^9^, ^10^, ^11^, ^12^, ^13^, ^14^, ^15^ ^]^ nonlinear effects,^[^ ^16^, ^17^, ^18^, ^19^, ^20^, ^21^, ^22^, ^23^, ^24^, ^25^ ^]^ and mechanical memory.^[^ ^26^, ^27^, ^28^, ^29^, ^30^ ^]^ The potential functionalities of acoustic metamaterials could be game‐changing for applications ranging from noise reduction^[^ ^31^, ^32^, ^33^, ^34^ ^]^ to acoustic cloaking.^[^ ^35^, ^36^, ^37^, ^38^ ^]^ Most of the existing literature on metamaterials considers fixed designs to control low‐amplitude harmonic waves. A growing trend in metamaterials research is to introduce tunability within the unit cell design. Such tunability can be achieved through external stimuli (e.g., magnetic,^[^ ^39^, ^40^, ^41^, ^42^, ^43^ ^]^ mechanical,^[^ ^44^, ^45^ ^]^ electric^[^ ^46^, ^47^, ^48^ ^]^ or thermal^[^ ^49^, ^50^ ^]^ input). In most cases, the tunability field or the external trigger is decoupled from the propagating wave, which might hinder its seamless integration as functional units or require converting signals from one domain to another. An alternative avenue could be harnessing the content of the wave (e.g., frequency, amplitude or both frequency and amplitude) to tune metamaterials. In this paper, we employ analytical, numerical, and experimental methods to design a dynamically tunable metamaterial capable of selectively manipulating mechanical waves based on their (1) frequency, (2) amplitude, or (3) a combination of frequency and amplitude. We present various scenarios demonstrating a metamaterial configuration capable of shifting between transmission and attenuation and vice versa. Such metamaterial can serve as a filter for both high and low amplitude signals. Furthermore, we demonstrate phase transitions occurring while maintaining the metamaterial's state of either attenuation or transmission. Such metamaterial can maintain their functionality–either wave attenuation or transmission–while also recording extreme events that trigger their transition (i.e., have memory).

## 2 Design Methodology

**Figure 1:**
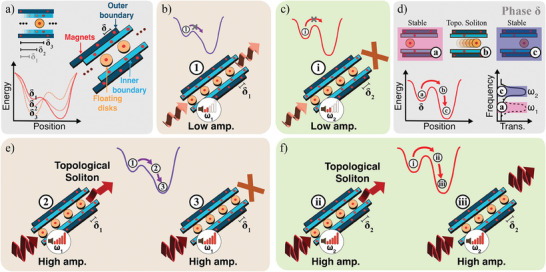
Concept.(a) Schematic of the proposed metamaterial composed of floated disks surrounded by two magnetic boundaries that can slide against each other in a programmable fashion resulting in a programmable potential‐energy landscape of the same unit cell with different boundary alignment δ. All magnets are aligned in the same direction, i.e., all are in repulsion. At low amplitude harmonic wave excitation the metamaterial can (b) allow waves to propagate or (c) stop waves from propagating depending on the incoming wave frequency. (d) For high amplitude wave excitation the disk position can flip from one stable configuration to the other, which changes the metamaterials frequency transmission range. The high amplitude excitation can change the metamaterial: (e) from transmitting the wave to attenuating it or (f) from attenuating the wave to transmitting it. Displacement in the x and y‐direction represent longitudinal and shear motion, respectively.

**Figure 2:**
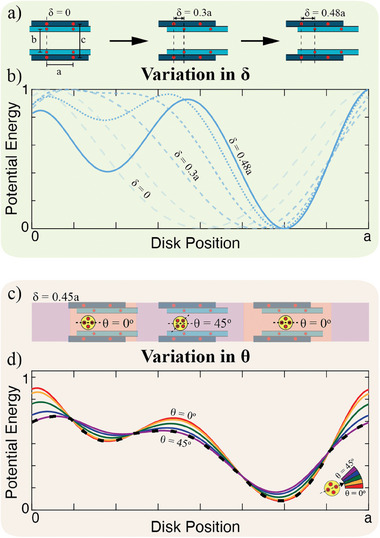
Energy landscapes. (a) Schematic of inner boundary shift. (b) Potential energy change in the unit cell as the outer boundary shifts. (c) Minimum energy orientation schematic of a four magnet disk traversing through the unit cell. (d) Potential energy of a disk varying orientation. Dashed black line represents the self‐oriented minimum energy state.

Our metamaterial is composed of free‐floating disks with embedded permanent magnets hovering over an air‐bearing table.^[^ ^51^, ^52^, ^53^, ^54^, ^55^, ^56^ ^]^ The disks are confined within a hard boundary of embedded permanent magnets. To intrinsically incorporate tunability within our design, we utilize two pairs of straight magnetic boundaries, an inner and an outer boundary. By controlling the separation distance between the magnets of the inner and outer boundaries, δ, we can engineer the energy landscape of the unit cell, and therefore change the resting position of the magnetic disk (**Figure** **1**a). Depending on where the disk sits within the unit cell dictates its dynamical characteristics including its wave filtering abilities. We consider a unit cell with a given length *a*, inner boundary vertical spacing *b*, and outer boundary vertical spacing *c* (**Figure** **2**a). While *a*, *b*, and *c* are fixed, the relative distance between the magnets in both boundaries, δ, can be tuned through a simple mechanical slide of either boundary. A change in δ, translates to a change in the energy landscape of the unit cell, *E*, which in turn shifts the minimum energy point where the disks rest (Figure 2b). The energy within the cell $E=\int_{0}^{a}-Fdx$, where *F* is the repulsion force between the permanent magnets within a single unit cell. At δ = 0, the energy landscape is symmetric and the minimum energy position lies at the center of the unit cell. As δ increases, the minimum energy position shifts to the right, however, the energy potential remains mono‐stable up to δ ⩽ 0.3*a*. As δ increases further, another energy local minimum nucleates to the left of the global minimum, due to the balance between the inner and the outer boundary forces, giving rise to bi‐stable potential wells. The energy landscape of these two potential wells dictates the amount of energy needed to push a disk from one well to the other. Once δ crosses 0.5*a*, the energy landscape is mirrored, i.e., the potential energy is the same for both δ = 0.4*a* and δ = 0.6*a* with the minimum energy position swapped from right to left.

Due to the lack of friction and physical constraints on the disks, the disks are free to self‐orient to minimize their own potential energy. For disks with one embedded magnet, the minimum energy position is a function of δ only and does not depend on the orientation of the disk. For disks with more than one embedded magnet (i.e., 2, 3, or 4 magnets per disk), disk orientation, θ, is crucial in determining the minimum energy position. In the case of four embedded magnets, the disk self‐orients to θ = 0° at the local and global minimum energy positions (Figure 2c,d). However, when the disk transitions from one minimum energy well to the other, it will self‐orient to a 45° angle as that is the lowest energy state when the disk is located between the energy wells.

## 3 Dispersion Analysis

To understand the dynamics of our proposed metamaterials, we investigate their wave propagation characteristics. We start our analysis by assuming an infinite repetition of a single unit cell in space. We use Bloch's theorem to obtain a correlation between wavenumber and frequency and predict the transmission frequency ranges for our metamaterials. Using a solution of Bloch's form to solve the equation of motion, we obtain an eigenvalue problem in the form: $\left[-\omega^{2}M+K\left(\kappa \right)\right]\phi =0$, where ω is frequency, **M** = *m* **I**_2 × 2_ is the mass matrix, **K**(κ) is the stiffness matrix, κ is the wavenumber, and **ϕ** = [*u*, *v*]^*T*^ is the displacement in the *x* and *y* directions, respectively.

(1) K=∑n=1N(∑η=12N[f′(dn,η)len,η⊗en,ηcos(κa)−1K=+f(dn,η)l(dn,η)(I−en,η⊗en,η)cos(κa)−1]K=−∑α=14f′(dn,α)sen,α⊗en,α+f(dn,α)s(dn,α)(I−en,α⊗en,α)K=−∑β=14f′(dn,β)len,β⊗en,β+f(dn,β)l(dn,β)(I−en,β⊗en,β))

where *N* is the number of magnets per disk, *f*(*d*) = *Ad* ^γ^ models the nonlinear magnetic repulsion between magnets, *f*′(*d*) is the repulsive force's first derivative, **e**_*i*, *j*_ is the unit vector from magnet *i* to magnet *j, s* and *d*_*i*, *j*_ is the distance between magnet *i* and magnet *j*. *n* is the index for disk magnets, η is the index of disk magnets in neighboring cells, α is the index for inner boundary magnets, β is the index for outer boundary magnets (See Figure S7, Supporting Information, for more details), *a* is the unit cell length, and ⊗ is the dyadic product. The smaller inner boundary magnets have a repulsive force $f{\left(d\right)}_{s}=A_{s}d^{\gamma_{s}}$ while the magnets in the outer boundaries and disks have repulsion force $f{\left(d\right)}_{l}=A_{l}d^{\gamma_{l}}$. Experimental test with a load cell result in $A_{s}=8.841\times 10^{-10}\;N/m^{\gamma_{s}}$, γ_*s*_ = −3.035, $A_{l}=9.1362\times 10^{-11}\;N/m^{\gamma_{l}}$, and γ_*l*_ = −4.

**Figure 3:**
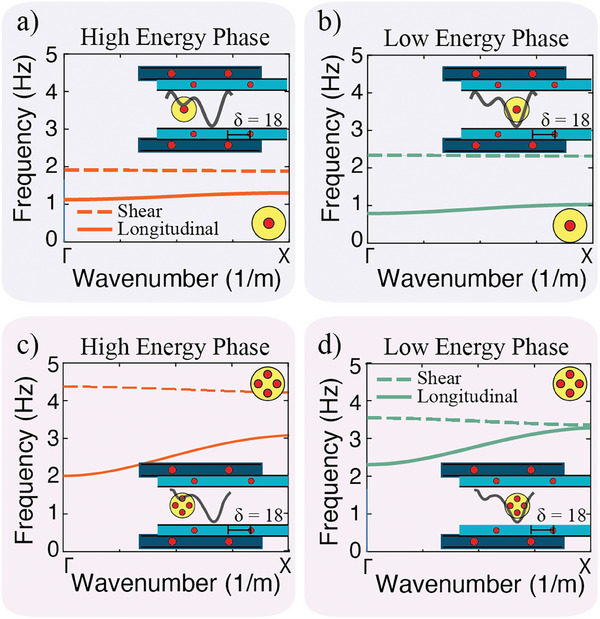
Dispersion curves. Analytical dispersion curves for metamaterial with one magnet per disk at the (a) higher energy potential well and (b) lower energy potential well at δ = 0.45*a* = 18mm. Analytical dispersion curves for metamaterial with four magnets per disk at the (c) higher energy potential well and (d) lower energy potential well. Solid lines represent longitudinal modes (vibrations in line with the metamaterial's *x* axis). Dashed lines represent shear modes (vibrations in line with the metamaterial's *y* axis). The inset in each panel shows the unit cell schematic with the position of the disk at θ = 0° overlaid on top of the unit cell potential energy diagram. The grey lines in the inset depicts the energy landscape.

There exists two stable unit cell configurations for certain δ values, where the disk can reside in either the lower or the higher energy potential well (Figure 2). Depending on where the disk resides, the magnetic stiffness will be different—as the distances between the disk magnets and the boundary magnets are different‐ and therefore the corresponding metamaterial's frequency transmission regions will differ. We calculate the dispersion curves for both disk positions but the same unit cell configuration (i.e., *a* = 40, *b* = 25, *c* = 39.5 and δ = 0.45*a* = 18 mm) (**Figure** **3**). In the case of a single magnet per disk, when the disk is in the higher energy position the longitudinal transmission frequency is from 1.1 to 1.3 Hz, and the shear transmission frequency is from 1.9 to 2.0 Hz (Figure 3a). However, when the disk is at the lower energy position *‐for the same unit cell parameters‐* the longitudinal transmission frequency changes to 0.8–1.0 Hz and the shear transmission frequency changes to 2.2–2.3 Hz (Figure 3b). In the case of four magnets per disk, the magnetic stiffness is higher, due to the additional embedded magnets within the disk. The higher stiffness translates to higher transmission frequency ranges. When the disk is in the higher energy position, the longitudinal transmission frequency is from 2.0 to 3.1 Hz, and the shear transmission frequency is from 4.2 to 4.3 Hz (Figure 3c). However, when the disk is at the lower energy position the longitudinal transmission frequency changes to 2.3–3.3 Hz and the shear transmission frequency changes to 3.4–3.6 Hz (Figure 3d).

**Figure 4:**
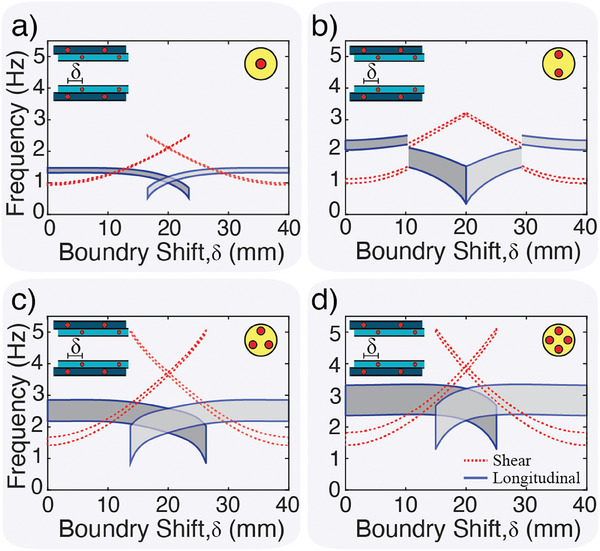
Tunability. Analytical predictions for metamaterial passbands as outer boundary is shifted. (a) One, (b) two, (c) three, and (d) four magnet per disk metamaterials tunablility. Blue solid lines represent bounds of longitudinal passbands. Red dashed lines represent bounds of shear passbands. Dark gray shading is when the disk is in the minimum energy state to the right of δ. Light gray shading is when the disk is in the minimum energy state to the left of δ.

To capture the effect of the outer‐boundary shift on the wave propagation characteristics of our metamaterials, we calculate the dispersion curves and plot the transmission frequency ranges as a function of δ. For every value of δ, we calculate the equilibrium position of the disk with one magnet (and both position and orientation angle θ for disks with more than one magnet). We use the resting position (and angle) to calculate the dispersion curves for each unit cell. It is worth noting that due to the disk rotational symmetry, a disk with four magnets at θ = 0°, 90°, 180°, and 270° is the same, acoustically. Therefore, theoretically speaking, adding the bi‐stable potential of the boundary to the disk rotational symmetry, one can anticipate multi‐stability. However, all these stable cases collapse into two acoustically unique states (i.e., bi‐stable). We vary the outer‐boundary position, δ, from 0 to *a* and obtain the corresponding dispersion curves for the resulting unit cell configurations (**Figure** **4**). For unit cells with two potential wells, we consider both stable configurations and plot both their transmission frequency ranges (Figure 4a,c,d). It is worth noting that the transmission ranges are mirrored about the unit cell midpoint due to symmetry. In the case of one, three, and four magnets per disk, the boundary shift has a relatively minute effect on the transmission frequency range for ≈δ ⩽ 10mm, particularly for longitudinal waves. As δ increases, the longitudinal transmission range shifts to lower frequencies, while the shear transmission range shrinks and shifts to higher frequencies. For boundary shifts ranging from ≈15 ⩽ δ ⩽ 25 mm, there are two stable positions for the disks to occupy with distinct transmission ranges for both shear and longitudinal excitation. In the case of two magnets per disk, the boundary shift has a gradual effect on the transmission frequency range for ≈δ ⩽ 10 mm, for both longitudinal and shear waves. At δ = 10.5 mm, a discontinuity in the transmission occurs because of a jump in the disk orientation angle from θ = 90° to θ = 0°. After the disk orientation changes, the transmission ranges of the shear and longitudinal modes flip, but continue to systematically shift upward (for shear) and downward (for longitudinal). It is worth noting that all the possible configurations for the two magnets per disk are mono‐stable (i.e., no bi‐stability) (Figure 4b).

## 4 Verification

**Figure 5:**
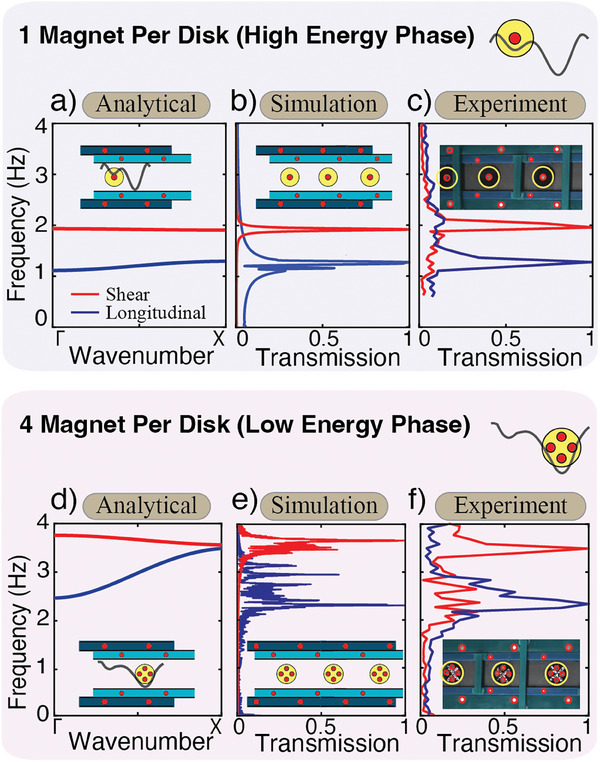
Model validation. Analytical passbands with frequency versus wave number for (a) 1 magnet per disk in the high energy phase and (d) 4 magnets per disk in the low energy phase. The inset shows the minimum energy landscape and differentiates the high and low energy phases. (b,e) FFT of simulated metamaterial under shear and longitudinal excitation. The inset shows a finite simulated metamaterial. (c,f) FFT of experimental metamaterial existed in the longitudinal and shear directions. Inset depicts the top down view of the metamaterial with disk highlighted in yellow and magnets highlighted in red.

In order to verify our infinite model assumption, we measure the response of finite metamaterials both numerically and experimentally. We start our numerical verification utilizing the Newmark method. We consider the sum of forces and moments to calculate the translation and rotation of two finite metamaterial samples with 10 disks each, one with a single embedded magnet per disk (**Figure** **5**a–c) and another with four embedded magnets per disk (Figure 5d–f). For the considered unit cell parameters (i.e., *a* = 40, *b* = 25, *c* = 39.5 and δ = 0.45*a* = 18 mm), there are two stable states, where the disk can be on either the left or the right minimum energy position. In the case of one embedded magnet per disk, all the disks are placed at the higher energy position, while in the case of the four magnets per disk, all the disks are at the lower energy position. We excite the metamaterial from one end with a chirp signal ranging from 0 to 10 Hz and measure the transmitted signal through the metamaterial. We apply the fast Fourier transform (FFT) to the transmitted signal to convert it to the frequency domain to compare it with the dispersion calculations. We define transmission as the FFT amplitude of the disk's displacement at the center of the lattice normalized by the FFT amplitude of the excited disk. We repeat the simulation twice, once with a shear excitation and another with a longitudinal one. We observe a very good agreement with our dispersion prediction and the numerically simulated transmission ranges for both cases.

To validate our analytical and numerical simulations, we realize and characterize our metamaterials experimentally. The disks are 3D printed out of Veroblack material with a diameter of 15 mm using the Connex printer. Each disk is embedded with either 1, 2, 3, or 4 cylindrical magnets with a diameter of 3 mm and a height of 3 mm. The boundaries are fabricated out of an acrylic glass sheet with 3 mm thickness using laser cutting (Full‐Spectrum 24 Pro‐Series). The outer boundary magnets have the same dimensions as the disk magnets, while the inner boundary magnets are cylindrical magnets with a diameter of 2 mm and a height of 1 mm. To minimize friction, ensuring the dominating force in the system is the magnetic force, the disks are suspended on an air bearing (NewWay S1345PA1500) which results in a damping ratio of ζ = 0.01. Glass slides were affixed to the bottom of each disk and fabric was added to the bottom of the acrylic boundary to ensure smooth sliding on the air bearing.

We excite the system using an electromagnet (type: kk‐P20/25, DC12V Kaka Electric) and a signal generator (type: Keysight Technologies 33512B). We excite the left most disk of the metamaterial magnetically with a chirp signal (0–10 Hz) in both the longitudinal and the shear directions, separately. We record the motion of the disks using a bird‐eye view camera (Blackfly S USB3). We post process the images using Digital Image Correlation software (DICe). We apply the fast Fourier transform (FFT) on the obtained displacements on a given disk to calculate the transmission frequencies for our metamaterials. We observe a very good agreement between the analytical dispersion, numerically simulated transmission ranges, and experimentally measured ones. It is worth noting that experimental shear excitation, in the case of the four magnets per disk, results in notable motion in the transmission range of the longitudinal mode. This could be due to an additional minor rotational and longitudinal excitation by the magnetic striker passing through the magnetic field of the four magnets of the disk, while being perpendicular to the metamaterial's periodicity direction.

## 5 Amplitude Dependency

Filtering harmonic waves based on their frequency is very desirable. Figures 3, 4, 5 demonstrate our metamaterials' ability to control waves based on their frequency. However, in many cases, filtering harmonic waves based on both amplitude and frequency is essential, particularly, in sound insulation and sensing applications (e.g., eliminating undesirable low or high amplitude noise in measurements). Given the bi‐stable nature of our metamaterials, they can exist in two different stable phases within the same geometry (i.e., boundary configuration). Moreover, the metamaterials can transition from one stable phase to the other, generating a topological soliton at specific amplitude threshold. Such transition in phase can change a passband frequency into a stopband one, and vice versa, which gives the metamaterials the ability to intrinsically discriminate between harmonic waves based on both their frequency and amplitude.

**Figure 6:**
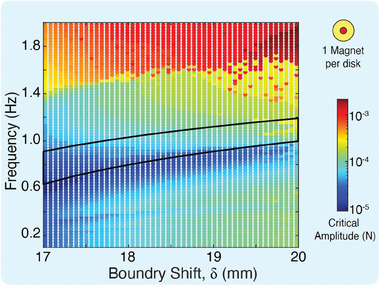
Critical Amplitude. Numerical simulations of critical amplitude over varying boundary configurations and excitation frequencies. Color bar represents minimum amplitude required to trigger topological soliton. Black line represent analytically predicted pass band for each boundary configuration.

To determine the critical amplitude that can cause our bi‐stable metamaterial to transition from one state to the other and nucleate a topological solution, we consider the case of a single magnet per disk. The metamaterial is bi‐stable for boundary parameter 17 mm ⩽ δ ⩽ 20 mm for the considered single magnet unit cell parameters (i.e. *a* = 40, *b* = 25, and *c* = 39.5 mm). The critical amplitude (the amplitude to transition from high energy state to low energy state) is a function of two parameters, the outer boundary configuration δ, and the excitation frequency ω. We utilize the Newmark method to simulate the transition waves and the nucleation of the topological solution. We iterated through each bi‐stable energy configuration, exciting the left most disk with ω ranging from 0 to 2 Hz, and a low enough amplitude that guarantees no transition. We repeat the simulations with increasing amplitudes until observing a topological soliton and denote the excitation amplitude as the critical amplitude for the δ − ω pair (**Figure** **6**).

The critical amplitude map shows a stark dependency on the frequency of excitation, spanning more than two orders of magnitude. We note a band of low critical amplitudes is required for initiating solitons near, but notably below, the analytically predicted passband (marked with black lines in Figure 6). This alludes to the fact that at or near the passband, lower amplitudes are needed to achieve a transition from high to low energy positions within the unit cell as resonance can accumulate energy within the system, which allows the disk to transition under less external excitation amplitude. Topological solitons are only triggered when the unit cell is oscillating at its maximum value within its minimum energy well. These large oscillations in our magnetic metamaterial are highly nonlinear and therefore the material exhibits nonlinear stiffness softening^[^ ^57^ ^]^ where the passband of the material drops in frequency as nonlinear effects take shape (See Appendix D). It is worth noting that the critical amplitudes shows a sharp increase for frequencies higher than 1.4 Hz. This amplitude cliff, distinctly marks high amplitude stopbands.

## 6 Control of Both Amplitude and Frequency

**Figure 7:**
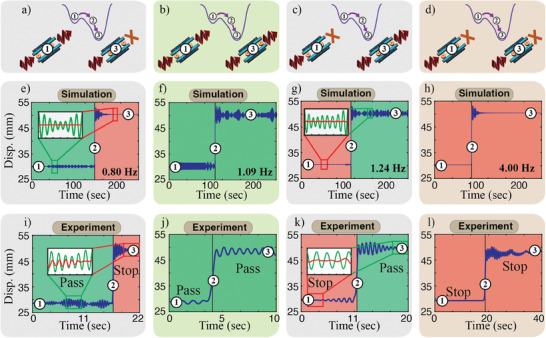
Functional topological solitons. (a–d) Conceptual schematic of a harmonic wave excitation leading to topological solitons resulting in a change within the metamaterial from the high energy to the low energy disk position. Numerical displacement profiles of a single disk at the center of the metamaterial showing transformation from (e) passband to stopband, (f) passband to passband (g) stopband to passband, and (h) stopband to stopband. Experimental displacement profiles of a single disk at the center of the metamaterial showing transformation from (i) passband to stopband, (j) passband to passband, (k) stopband to passband, and (l) stopband to stopband.

To demonstrate our metamaterials' ability to control harmonic waves based on both their frequency and amplitude, we consider four different scenarios using the same metamaterial with different transformations: (1) from passband to a stopband (**Figure** **7**a), (2) from passband to a passband (Figure 7b), (3) from stopband to a passband (Figure 7c), and (4) from stopband to a stopband (Figure 7d). In all cases, we excite the metamaterials from the left‐most end with a single frequency excitation. The amplitude of the excitation increases in time until it reaches a critical amplitude, *A* = *A*_critical_, at which a topological soliton nucleates causing a phase transition. The forcing function, *F*, is defined as $F\left(t\right)=A\cos\left(\omega t\right)\left(e^{-\frac{t}{\tau }}-1\right)$ where *A* is the input amplitude, ω is the input frequency, and τ represents the amplitude growth rate, dictating how soon *A*_critical_ is reached. Once *A*_critical_ is reached, it is sustained throughout the rest of the simulation. In all four cases, the considered unit cell parameters are (i.e., *a* = 40, *b* = 25, *c* = 39.5 and δ = 0.475*a* = 19 mm) and each disk has one embedded magnet. A value of τ ≈ 125 sec is used to reach critical amplitude (and phase change) halfway through the simulation. The transmission range for the metamaterial at high energy phase is 0.79–1.12 Hz, while at the low energy phase the transmission is 1.03–1.30 Hz for longitudinal waves.

In the first considered case, (1), transitioning from passband to stopband phases, we excite our metamaterial at 0.8 Hz, which is a transmission frequency at the high energy phase. At low amplitude excitation (i.e., below *A*_critical_), the wave propagates through the material, but does not cause phase transition, per design. Once *A*_critical_ is reached, a topological soliton nucleates and the metamaterial changes phase (i.e., low energy phase). The new emerging phase does not allow the excitation frequency of 0.8 Hz to propagate as it now falls within a band gap frequency (Figure 7e). The inset in Figure 7e, shows the stark difference between the transmission amplitude of the wave through the metamaterial before and after the phase transition. In case (2), the metamaterial experiences a phase change, but without changing its transmission at the excitation frequency of 1.09 Hz (i.e., from passband to passband). At first, the wave propagates without the nucleation of the topological soliton, due to its low amplitude. However, once *A*_critical_ is reached, the metamaterial transitions into the low energy phase, while sustaining the propagation of the harmonic wave (Figure 7f). Case (2) might seem trivial, because the wave ultimately continues to transmit thought the metamaterial. However, such property can serve a memory functionality, where the material can “remember” being excited with an amplitude exceeding its *A*_critical_.

In the third considered case (3), transitioning from stopband to passband, we excite our metamaterial at 1.24 Hz. At low amplitude excitation (i.e., below *A*_critical_), the wave does not propagate through the material, and also does not cause phase transition, per design. Again, once *A*_critical_ is reached, a topological soliton nucleates and the metamaterial changes phase (i.e., low energy phase). The new emerging phase supports the propagation of the excitation frequency of 1.24 Hz as it falls within a passband frequency (Figure 7g). The inset in Figure 7g, shows the stark difference between the transmission amplitude of the wave through the metamaterial before and after the phase transition. In the last case (4), the metamaterial experiences a phase change, but without changing its attenuation state at the excitation frequency of 4 Hz (i.e., from stopband to stopband). Both before and after the nucleation of the topological soliton, the wave does not propagate through the metamaterial (Figure 7h). Case (4) might again seem trivial, because the wave never transmits thought the metamaterial. However this can also exhibit the memory functionality where the material “remembers” being excited with an amplitude exceeding its *A*_critical_. It is worth noting that *A*_critical_ is not a constant value for a given δ, but it is rather a function of frequency (See Section 5). The critical amplitudes for the four considered cases are *A*_1_ = 5.1 × 10^−5^ N, *A*_2_ = 1.0 × 10^−4^ N, *A*_3_ = 1.0 × 10^−4^ N, and *A*_4_ = 1.3 × 10^−2^ N.

To further validate our metamaterials' utility in controlling harmonic waves based on both their frequency and amplitude, we *experimentally* test the same four scenarios. It is worth noting that the excitation amplitudes used in the experiments are fixed, in contrast to the numerical simulations, where we increase the amplitude gradually to reach *A*_critical_. Starting with amplitudes close to *A*_critical_ in our experiments does not change any of the considered physics, but relatively shortens the experiment time, which overcomes the limited capacity of the storage memory of the high speed cameras at the captured time resolution. We excite our metamaterials at the same four frequencies considered numerically and observe a clear matching in behaviour with our numerical simulations. Furthermore, we experimentally observe the same numerically recorded variation in *A*_critical_ as a function of the excitation frequency within the same metamaterial configuration (Figure 7i–l). We note that both numerical and experimental validations take place at ultra‐low frequencies within the infrasound range (i.e., below 20 Hz). Such frequency range requires conventional metamaterials to span meters in length or register kilograms in weight to be able to control infrasound waves. Our metamaterials can access such range with a few centimeter in length and a few grams in weight. These metamaterials could potentially be used as infrasound sensors with intrinsic memory.

## 7 Conclusion

In conclusion, we use analytical, numerical, and experimental methods to investigate the utility of metamaterials in controlling harmonic waves based on both amplitude and frequency. Our metamaterials are composed of free floating disks with embedded permanent magnets confined within a magnetic boundary. By programming the boundary's magnetic field, we can change the energy landscape of the metamaterials and the equilibrium positions of the disks. Both disk position and surrounding magnetic field dictate the stability of the unit cell and its transmission characteristics. By engineering the metamaterials to retain bi‐stable configurations (i.e., two stable phases), we elucidate the required conditions for a transition wave (i.e., a topological soliton) to nucleate causing a phase change within the metamaterial. Each of these phases has its own transmission frequency range. We harness this phase change to control harmonic waves based on their amplitude and frequency. We showcase different scenarios with the same metamaterial configuration that can phase change from transmission to attenuation and vice versa. Such material can be utilized as a filter to either high or low amplitude signals. In addition, we show phase transitions taking place while preserving the metamaterial's state of attenuation or transmission. Such metamaterials can continue their functionality (i.e., either attenuation or transmission of waves) while keeping a record of extreme events that can cause their transition. Our metamaterials can open the door for the next generations of advanced and functional acoustic devices.

## Conflict of Interest

The authors declare no conflict of interest.

## Supporting information

Supporting Information

## Data Availability

The data that support the findings of this study are available from the corresponding author upon reasonable request.

## References

[1] P. A. Deymier , Acoustic Metamaterials and Phononic Crystals , vol. 173, Springer Science & Business Media, 2013.

[2] S. A. Cummer , J. Christensen , A. Alù , Nat. Rev. Mater. 2016, 1, 1.

[3] G. Ma , P. Sheng , Sci. Adv. 2016, 2, e1501595. 26933692 10.1126/sciadv.1501595 PMC4771441

[4] D. L. Sounas , C. Caloz , A. Alu , Nat. Commun. 2013, 4, 2407. 23994940 10.1038/ncomms3407

[5] N. Nadkarni , A. F. Arrieta , C. Chong , D. M. Kochmann , C. Daraio , Phys. Rev. Lett. 2016, 116, 244501. 27367390 10.1103/PhysRevLett.116.244501

[6] C. Coulais , D. Sounas , A. Alu , Nature 2017, 542, 461. 28192786 10.1038/nature21044

[7] A. P. Browning , F. G. Woodhouse , M. J. Simpson , Proc. Roy. Soc. A 2019, 475, 20190146. 31423095 10.1098/rspa.2019.0146 PMC6694314

[8] B. Deng , M. Zanaty , A. E. Forte , K. Bertoldi , Phys. Rev. Appl. 2022, 17, 014004.

[9] N. Nadkarni , C. Daraio , D. M. Kochmann , Phys. Rev. E 2014, 90, 023204. 10.1103/PhysRevE.90.023204 25215840

[10] S. Katz , S. Givli , Extreme Mechanics Letters 2018, 22, 106.

[11] Y. Zhang , B. Li , Q. Zheng , G. M. Genin , C. Chen , Nat. Commun. 2019, 10, 5605. 31811130 10.1038/s41467-019-13546-y PMC6898320

[12] M. Hwang , A. F. Arrieta , Sci. Rep. 2018, 8, 3630. 29483610 10.1038/s41598-018-22003-7 PMC5827759

[13] L. Jin , R. Khajehtourian , J. Mueller , A. Rafsanjani , V. Tournat , K. Bertoldi , D. M. Kochmann , Proc. Natl. Acad. Sci. 2020, 117, 2319. 31969454 10.1073/pnas.1913228117 PMC7007517

[14] H. Yasuda , L. Korpas , J. Raney , Phys. Rev. Appl. 2020, 13, 054067.

[15] N. Vasios , B. Deng , B. Gorissen , K. Bertoldi , Nat. Commun. 2021, 12, 695. 33514707 10.1038/s41467-020-20698-9 PMC7846611

[16] B.‐I. Popa , S. A. Cummer , Nat. Commun. 2014, 5, 3398. 24572771 10.1038/ncomms4398

[17] J. R. Raney , N. Nadkarni , C. Daraio , D. M. Kochmann , J. A. Lewis , K. Bertoldi , Proc. Natl. Acad. Sci. 2016, 113, 9722. 27519797 10.1073/pnas.1604838113 PMC5024640

[18] X. Fang , J. Wen , B. Bonello , J. Yin , D. Yu , Nat. Commun. 2017, 8, 1288. 29101396 10.1038/s41467-017-00671-9 PMC5670230

[19] R. Khajehtourian , D. M. Kochmann , Extreme Mechanics Letters 2020, 37, 100700.

[20] B. Deng , P. Wang , V. Tournat , K. Bertoldi , J. Mech. Phys. Solids 2020, 136, 103661.

[21] H. Nassar , B. Yousefzadeh , R. Fleury , M. Ruzzene , A. Alù , C. Daraio , A. N. Norris , G. Huang , M. R. Haberman , Nat. Rev. Mater. 2020, 5, 667.

[22] B. Deng , P. Wang , Q. He , V. Tournat , K. Bertoldi , Nat. Commun. 2018, 9, 3410. 30143618 10.1038/s41467-018-05908-9 PMC6109112

[23] R. K. Pal , J. Vila , M. Leamy , M. Ruzzene , Phys. Rev. E 2018, 97, 032209. 29776120 10.1103/PhysRevE.97.032209

[24] Y. Chen , X. Li , G. Hu , M. R. Haberman , G. Huang , Nat. Commun. 2020, 11, 3681. 32704039 10.1038/s41467-020-17529-2 PMC7378557

[25] Y. Tang , Y. Chi , J. Sun , T.‐H. Huang , O. H. Maghsoudi , A. Spence , J. Zhao , H. Su , J. Yin , Sci. Adv. 2020, 6, eaaz6912. 32494714 10.1126/sciadv.aaz6912 PMC7209986

[26] H. Yasuda , T. Tachi , M. Lee , J. Yang , Nat. Commun. 2017, 8, 962. 29042544 10.1038/s41467-017-00670-w PMC5714951

[27] S. M. Montgomery , S. Wu , X. Kuang , C. D. Armstrong , C. Zemelka , Q. Ze , R. Zhang , R. Zhao , H. J. Qi , Adv. Funct. Mater. 2021, 31, 2005319.

[28] T. Chen , M. Pauly , P. M. Reis , Nature 2021, 589, 386. 33473228 10.1038/s41586-020-03123-5

[29] H. Han , V. Sorokin , L. Tang , D. Cao , Mechanical Syst. Sign. Process. 2023, 188, 110033.

[30] J. E. Pechac , M. J. Frazier , Appl. Phys. Lett. 2023, 122, 21.

[31] J. Zhu , J. Christensen , J. Jung , L. Martin‐Moreno , X. Yin , L. Fok , X. Zhang , F. Garcia‐Vidal , Nat. Phys. 2011, 7, 52.

[32] S. Kumar , H. P. Lee , in Acoustics , vol. 1, MDPI, 2019, pp. 590–607.

[33] N. Gao , Z. Zhang , J. Deng , X. Guo , B. Cheng , H. Hou , Adv. Mater. Technol. 2022, 7, 2100698.

[34] L. Y. L. Ang , F. Cui , K.‐M. Lim , H. P. Lee , Sustainability 2023, 15, 4113.

[35] H. Chen , C. T. Chan , Appl. Phys. Lett. 2007, 91, 18.

[36] D. Torrent , J. Sánchez‐Dehesa , New J. Phys. 2008, 10, 063015.

[37] H. Chen , C. T. Chan , J. Phys. D: Appl. Phys. 2010, 43, 113001.

[38] R. V. Craster , S. Guenneau , Acoustic Metamaterials: negative Refraction, Imaging, Lensing and Cloaking , vol. 166, Springer Science & Business Media, 2012.

[39] Y. Chen , X. Li , H. Nassar , A. N. Norris , C. Daraio , G. Huang , Phys. Rev. Appl. 2019, 11, 064052.

[40] F. Allein , V. Tournat , V. Gusev , G. Theocharis , Appl. Phys. Lett. 2016, 108, 161903.

[41] Z. Wang , Q. Zhang , K. Zhang , G. Hu , Adv. Mater. 2016, 28, 9857. 27654019 10.1002/adma.201604009

[42] J. O. Vasseur , O. B. Matar , J. Robillard , A.‐C. Hladky‐Hennion , P. A. Deymier , AIP Adv. 2011, 1, 041904.

[43] J.‐F. Robillard , O. B. Matar , J. O. Vasseur , P. A. Deymier , M. Stippinger , A.‐C. Hladky‐Hennion , Y. Pennec , B. Djafari‐Rouhani , Appl. Phys. Lett. 2009, 95, 124104.

[44] P. Wang , F. Casadei , S. Shan , J. C. Weaver , K. Bertoldi , Phys. Rev. Lett. 2014, 113, 014301. 25032927 10.1103/PhysRevLett.113.014301

[45] M. Kheybari , C. Daraio , O. R. Bilal , Appl. Phys. Lett. 2022, 121, 081702.

[46] J. Cha , C. Daraio , Nat. Nanotechnol. 2018, 13, 1016. 30201989 10.1038/s41565-018-0252-6

[47] M. Roshdy , T. Chen , S. Nakhmanson , O. R. Bilal , Extreme Mechanics Letters 2023, 59, 101966.

[48] M. Roshdy , O. R. Bilal , Adv. Mater. Technol. 2024, 2301562.

[49] K.‐C. Chuang , X.‐F. Lv , D.‐F. Wang , Appl. Phys. Lett. 2019, 114, 051903.

[50] K.‐C. Chuang , X.‐F. Lv , Y.‐H. Wang , J. Appl. Phys. 2019, 125, 055101.

[51] A. A. Watkins , O. R. Bilal , Front. Mater. 2020, 7, 606877.

[52] E. Norouzi , A. A. Watkins , O. R. Bilal , Phys. Rev. E 2021, 104, 044902. 34781554 10.1103/PhysRevE.104.044902

[53] A. A. Watkins , A. Eichelberg , O. R. Bilal , Phys. Rev. B 2021, 104, L140101.

[54] A. A. Watkins , A. Eichelberg , O. R. Bilal , Phys. Rev. Appl. 2022, 17, 024036.

[55] A. Eichelberg , A. A. Watkins , O. R. Bilal , Phys. Rev. Appl. 2022, 18, 054049.

[56] M. M. Samak , O. R. Bilal , APL Mater. 2024, 12, 1.

[57] H. Cho , B. Jeong , M.‐F. Yu , A. F. Vakakis , D. M. McFarland , L. A. Bergman , Int. J. Solids Struct. 2012, 49, 2059.

