# Supplementary material for: Bi‐Stable Metamaterials with Intrinsic Memory for Selective Wave Filtering Based on Frequency and Amplitude
Source: Adv Sci (Weinh). 2024 Nov 6;12(1):2405146. doi: 10.1002/advs.202405146 (PMC11714203; doi:10.1002/advs.202405146)
Supplement: Supplementary file 1 — Supporting Information [file ADVS-12-2405146-s001.pdf]

ADVANCED SCIENCE
Open Access

Supporting Information

for *Adv. Sci.*, DOI 10.1002/advs.202405146

Bi-Stable Metamaterials with Intrinsic Memory for Selective Wave Filtering Based on Frequency and Amplitude

*Nathan N. Stenseng, Mahmoud M. Samak and Osama R. Bilal**

Supplementary Information: Bi-stable Metamaterials with Intrinsic Memory for Selective Wave Filtering Based on Frequency and Amplitude

Nathan N. Stenseng, Mahmoud M. Samak, and Osama R. Bilal*
School of Mechanical, Aerospace, and Manufacturing Engineering, University of Connecticut, Storrs, USA

Appendix A: Numerical Concept Validation

In Figure 1b and 1e in the main manuscript, we propose the idea of a metamaterial that allows for the propagation of one frequency at low amplitude but attenuate the same frequency at higher amplitude (Phase A). Adjusting the material to a different setting (Phase B) should result in the opposite: attenuating the same frequency at low amplitude (Fig. 1c) while allowing its propagation at higher amplitude (Fig. 1f). Figure S1 depicts this in our metamaterial. A 1.24 Hz longitudinal displacement with low amplitude input excitation of 2 mm is prescribed to the left most disk of a 10 unit cells metamaterial (Fig. S1a). The wave passes through the metamaterial to the right most disk. When the same phase metamaterial experiences a high amplitude signal (13 mm), the signal is drastically attenuated (Fig. S1c). However, when the metamaterial is in Phase B, the opposite occurs. The 1.24 Hz longitudinal displacement with low amplitude of 2 mm is attenuated through the metamaterial (Fig. S1b) and the 13 mm high amplitude signal transmits through the metamaterial (Fig. S1d).

In addition to demonstrating the utility of our metamaterials at a single frequency with different amplitudes and phases (Fig. S1), we show a single metamaterials' design with the ability to control waves both based on their amplitude and frequency. We excite our metamaterial with a combination of high amplitude frequency, ω_{high} and low amplitude frequency, ω_{low}. Each of these frequencies is excited with two different amplitudes: high amplitude excitation, A_{high} and low amplitude excitation A_{low}. In the first demonstration, the metamaterial only admits the wave excited at ω_{low} with A_{low} (green signal in Figure S2a). All three other combinations, namely ω_{low} excited with A_{high}, ω_{high} excited at both A_{low} and A_{high}, do not propagate through the metamaterial (red signals in Figure S2a). In the second demonstration, the metamaterial only admits the wave excited at ω_{high} with A_{low} (green signal in Figure S2b). All three other combinations, namely ω_{low} excited with A_{low}, ω_{low} excited at A_{high} and ω_{high} excited at A_{high}, do not propagate through the metamaterial (red signals in Figure S2b). In the third demonstration, the metamaterial only admits the wave excited at ω_{low} with A_{high} (green signal in Figure S2c). All three other combinations, namely ω_{low} excited with A_{low}, ω_{high} excited at both A_{low} and A_{high}, do not propagate through the metamaterial (red signals in Figure S2c). In the fourth demonstration, the metamaterial only admits the wave excited at ω_{high} with A_{high} (green signal in Figure S2d). All three other combinations, namely ω_{low} excited with either A_{low} or A_{high}, and ω_{high} excited at A_{low}, do not propagate through the metamaterial (red signals in Figure S2d). These four demonstrations with 16 different cases, where we excite the same metamaterial (with the same δ) with different frequencies and amplitudes constitute an evidence to the utility of our metamaterial to selectively admit a specific combination of amplitude and frequency, by design, through the metamaterial (green signals) and block any other combination (plotted in red).

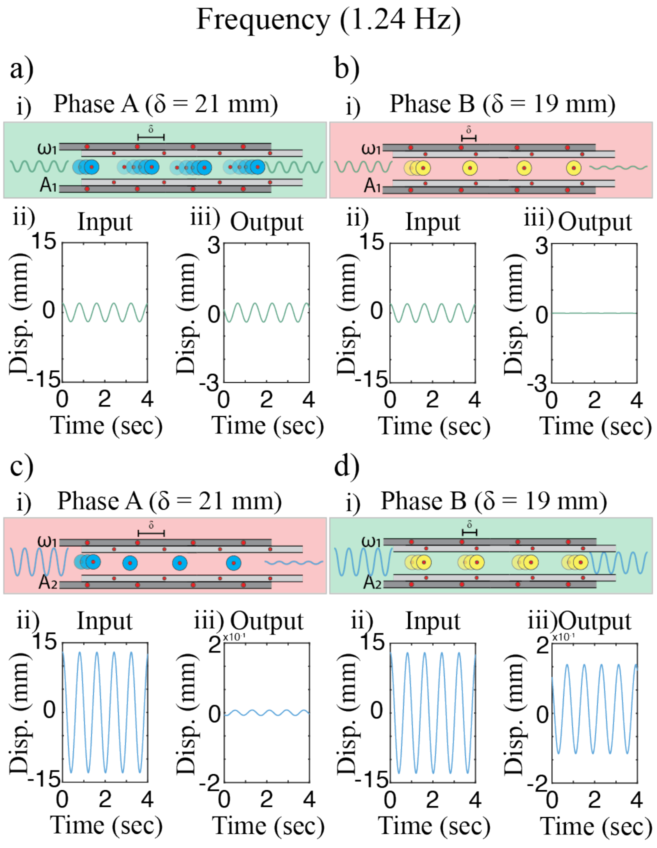

FIG. S1. **Concept Validation: Same Frequency**. Amplitude and frequency dependency for a single frequency through changing metamaterial phase. (a,c) Phase A, Low amplitude passband/high amplitude stopband. (b,d) Phase B, Low amplitude stopband/high amplitude passband. (i) Metamaterial phase and motion depicted with created input signal (ii) and measured output signal (iii).

* osama.bilal@uconn.edu

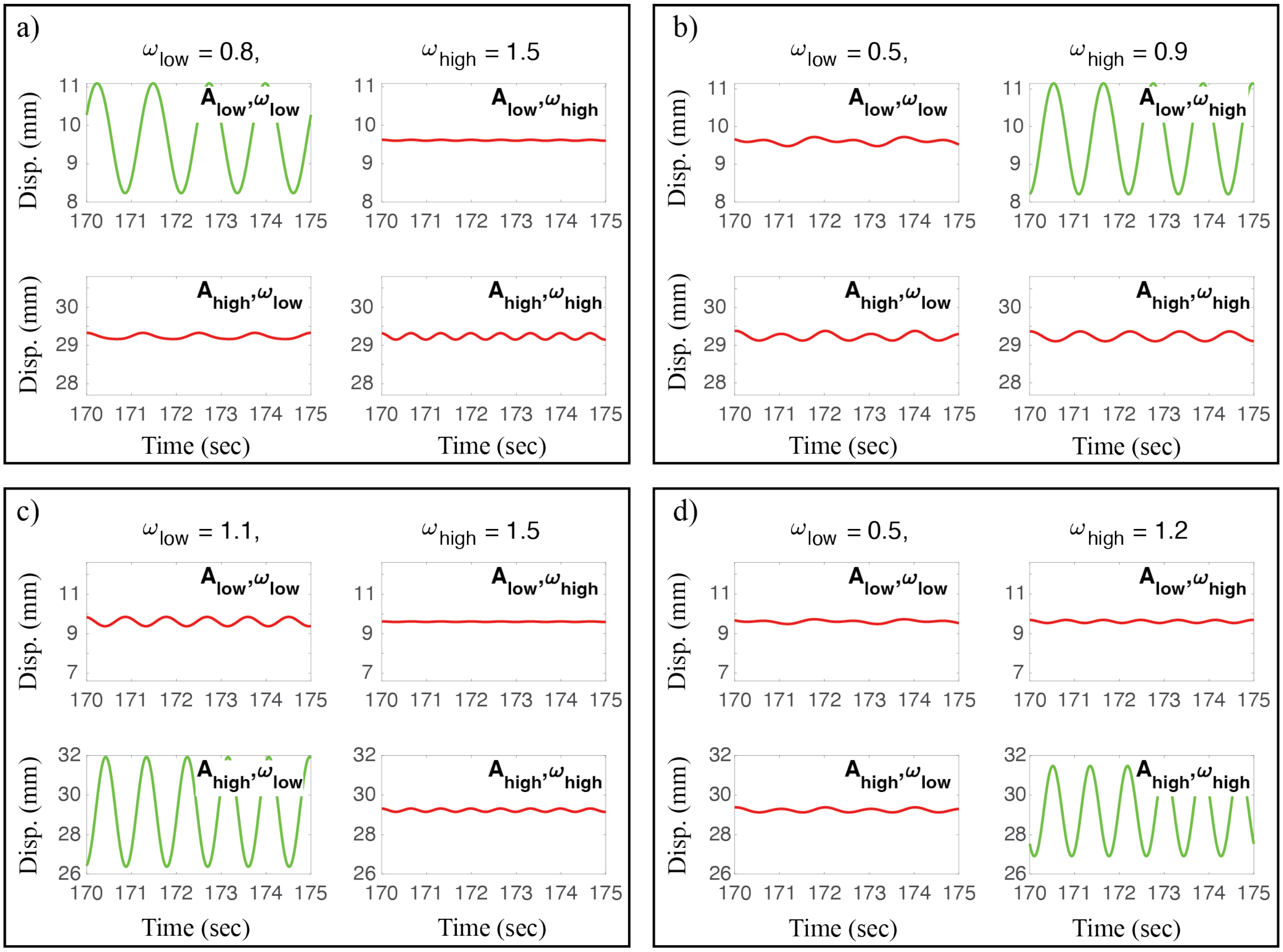

FIG. S2. **Concept Validation: Same Phase**. Transmitted waves through the same metamaterial with different combinations of amplitude and frequency excitations. Transmission only occurs (green lines) for waves with: (a) low amplitude/low frequency, (b) low amplitude/high frequency, (c) high amplitude/low frequency, and (d) high amplitude/high frequency. All other combinations are not allowed to propagate (red lines).

Appendix B: Multi-magnet Minimum Energy Positions

In our design methodology, our objective is to determine the position and orientation (state) of the disk within the unit cell. For all cases except those with two magnets per disk, we observe a minimum energy orientation of $\theta = 0°$. We find this by systematically varying the outer boundary shift (δ) and the disk's position within the unit cell (x) to identify the energy landscape, and therefore the minimum energy position for all metamaterial phases. Figure S3 shows the minimum energy disk position (x) corresponding to different boundary shift (δ). Cases where two disk positions are present for a given δ are bi-stable cases (i.e. the landscape has 2 minimum energy positions), where as the other majority of δ are mono-stable cases. The emergence of a new minimum energy state is represented by black dashed lines. For one magnet per disk, bi-stable energy states occur between 17 mm $\leq \delta \leq$ 23 mm. All minimum energy positions are obtained by sweeping through disk orientation angle θ for any disk with more than one embedded magnet. In the two magnets per disk case, we see no bi-stable states. However, the minimum energy position jumps from one half of the unit cell to the other at $\delta = 20$ mm. We note that at exactly $\delta = 20$ mm, there are two stable states, however, the transmission is not different with the disk on the right or the left of the inner boundary magnet (See Fig. 4b). Three magnets per disk has the largest bi-stable band between 14 mm $\leq \delta \leq$ 26 mm. It is worth noting that a disk with three magnets can be frustrated [53], exhibiting more intriguing stability dynamics. Lastly, four magnets per disk has bi-stable configurations between 15 mm $\leq \delta \leq$ 25 mm. While mono-stable metamaterial configurations still act as traditional filters in the frequency domain, incorporating bi-stable phases becomes crucial to address amplitude dependency in our metamaterial design.

Appendix C: Two Magnets Per Disk

As stated earlier, the two magnets per disk metamaterial has no utilizable bi-stable configurations. These findings are justified analytically in Figure S4. For comparative purposes, the four magnets per disk case is shown (Fig. S4c, d) in both a mono-stable phase $\delta = 10$ mm, and a bi-stable phase $\delta = 15$ mm. In both cases, there is little difference between disk orientation when scaled to the total energy landscape but the inset in Figure S4c clearly shows a minimum energy orientation of $\theta = 0°$. The difference in orientation energy is more abundantly clear in the $\delta = 15$ mm phase where the second energy position begins for four magnets per disk. Additionally, the experiments with minimal to no-friction utilizing the air bearing clearly show the validity of the analytically predicted minimum energy orientations in all unit cells along the metamaterial over all other angular orientations, regardless of the starting position of the disks. For two magnets per disk metamaterials, looking at the $\theta = 90°$ orientation (purple lines in Figure S4) match the energy landscapes of four magnets per disk. However $\theta = 0°$ has a much lower energy and is the preferred state of the free floating disks within the metamaterial. Additionally, unlike other disk configurations, at $\delta = 10$ mm, the two magnets per disk has a lower energy position when shifted to the right and oriented at $\theta = 90°$ (inset: purple), as compared to the $\theta = 0°$ orientation (inset: red).

Appendix D: Nonlinear stiffness softening

Section V in the manuscript contains a map of the critical amplitudes required to trigger a topological soliton for different excitation frequencies and metamaterial configurations. For linear metamaterials, the material's passband should have the lowest required input excitation to produce a resonance; however we experience the lowest critical amplitudes just below the analytically predicted and verified passband. This is due to nonlinear softening [57]. Softening takes place when nonlinear structures experience a lowering in transmission frequencies.

Figure S5c shows the analytical predictions for a single magnet per disk metamaterial with unit cell configuration $a = 40, b = 25, c = 39.5$, and $\delta = 18$ mm in the higher energy potential well. Next, we simulate a chirp excitation at an amplitude far lower than the critical amplitude, matching the verified results of Figure 5 in the main paper. However, Figure S5e shows the fast Fourier transform (FFT) of a chirp signal just below the amplitude required to trigger a topological soliton. This

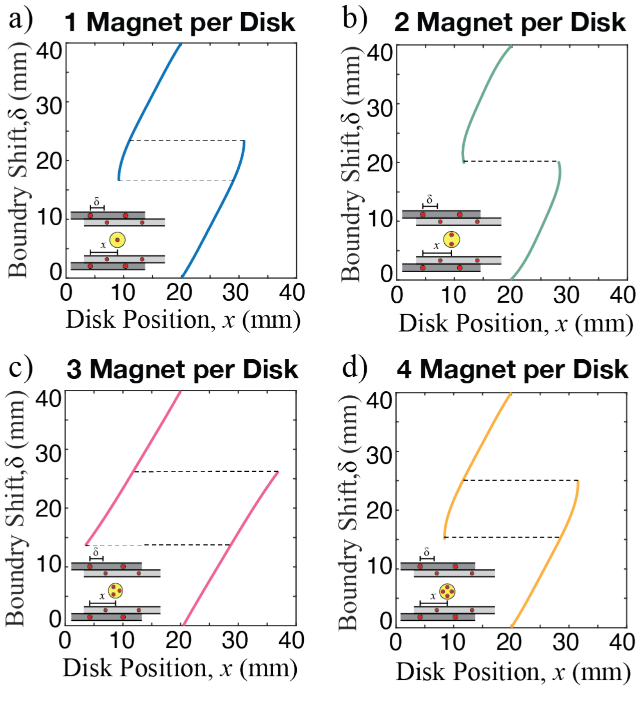

FIG. S3. **Minimum Energy Position**. Analytical calculation of minimum energy position within the unit cell for 1-4 magnets per disk (a-d). Region between black dashed lines represent the emergence of a second minimum energy positions. The inset depicts a schematic of the different disk in the unit cell.

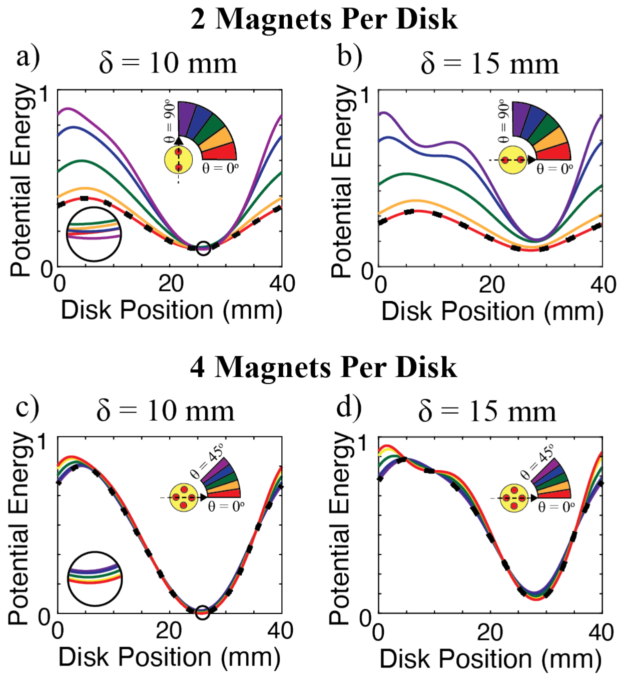

FIG. S4. **Two Magnets per Disk**. Analytical energy landscapes as disk orientation θ is varied (lines red - purple). Colored lines represent different orientations, reference color key inset in each figure. Two magnets disk at two mono-stable configurations, $\delta = 10$ mm (a) and $\delta = 15$ mm (b). Four magnets per disk mono-stable energy landscape (c) and bi-stable energy landscape (d) with bi-stable minimum energy positions at $x = 28$ mm and $x = 9$ mm

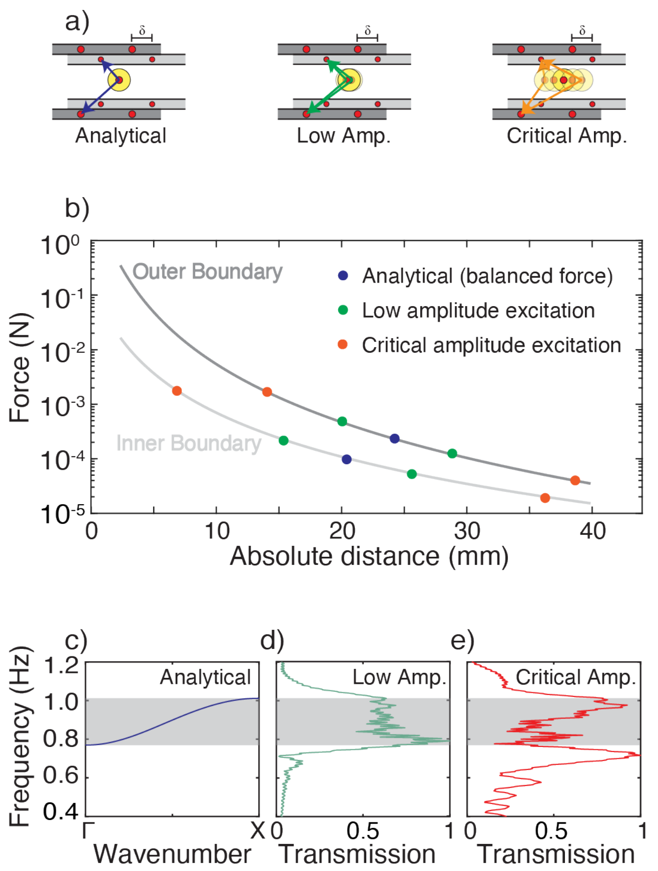

FIG. S5. **Nonlinear Stiffness Softening**. (a) Unit cell schematic with force vectors for analytical, low amplitude, and critical amplitude excitation. (b) Force-displacement graph of both the inner and outer boundary with dots marking the range of amplitudes for low and critical amplitude excitation. (c) Analytical passbands with frequency versus wave number. (d) FFT of simulated low amplitude signal frequency versus normalized amplitude. (e) FFT of simulated signal near critical amplitude. Grey box shows analytical passband predictions.

results in the most oscillation within the higher energy potential well. Under these highly nonlinear oscillations, we see softening in the material. The FFT has shifted below the analytically predicted passband (grey shaded region). Figure S5b shows the force-displacement graph of the disk with balanced forces in the considered unit cell, the displacement range for low amplitude and critical amplitude excitation.

Appendix E: Appendix: Experimental Setup

To experimentally verify our analytical and numerical calculations, we use the experimental setup presented in Figure S6. Panel S6a shows the optical table with the air-bearing mounted on it. The metamaterial is resting on top of the air-bearing with "top-down" lights and

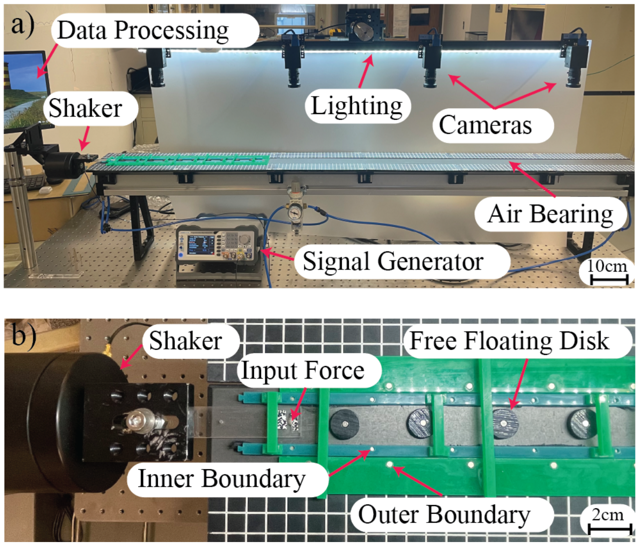

FIG. S6. **Experimental Setup**. (a) Laboratory setup of the metamaterial resting atop the air bearing. The shaker excites the system in the longitudinal direction using a signal generator. Lighting is added for the high speed cameras to record motion. (b) Zoom-in view of the metamaterial excited with a magnet as the input force attached to the shaker. The metamaterial consist of free floating disks, an inner boundary, and outer boundary, all of which have embedded magnets in repulsion.

cameras. The function generator is connected to an excitation source. The excitation in picture is set for the longitudinal direction. We record the motion of the disks using a bird-eye view high speed camera (Blackfly S USB3). We then post process the image using the digital image correlation engine (DICe). With the acquired position data, we apply the fast Fourier transform (FFT) to find the frequencies each disk is oscillating at.

Appendix F: Labeling and Calculations

The stiffness matrix, which is written in the main manuscript, is repeated here for convenience:

$$
\begin{aligned}
\mathbf{K} = \sum_{n=1}^{N}\Bigg(\sum_{\eta=1}^{2N}\Big[& f'(d_{n,\eta})_l \mathbf{e}_{n,\eta}\otimes\mathbf{e}_{n,\eta}\left[\cos(\kappa a)-1\right] \\
& + \frac{f(d_{n,\eta})_l}{(d_{n,\eta})}(\mathbf{I}-\mathbf{e}_{n,\eta}\otimes\mathbf{e}_{n,\eta})\left[\cos(\kappa a)-1\right]\Big] \\
- \sum_{\alpha=1}^{4}\Big[& f'(d_{n,\alpha})_s \mathbf{e}_{n,\alpha}\otimes\mathbf{e}_{n,\alpha} + \frac{f(d_{n,\alpha})_s}{(d_{n,\alpha})}(\mathbf{I}-\mathbf{e}_{n,\alpha}\otimes\mathbf{e}_{n,\alpha})\Big] \\
- \sum_{\beta=1}^{4}\Big[& f'(d_{n,\beta})_l \mathbf{e}_{n,\beta}\otimes\mathbf{e}_{n,\beta} + \frac{f(d_{n,\beta})_l}{(d_{n,\beta})}(\mathbf{I}-\mathbf{e}_{n,\beta}\otimes\mathbf{e}_{n,\beta})\Big]\Bigg)
\end{aligned}
$$

where N is the number of magnets per disk, $f(d) = Ad^{\gamma}$ models the nonlinear magnetic repulsion between mag-

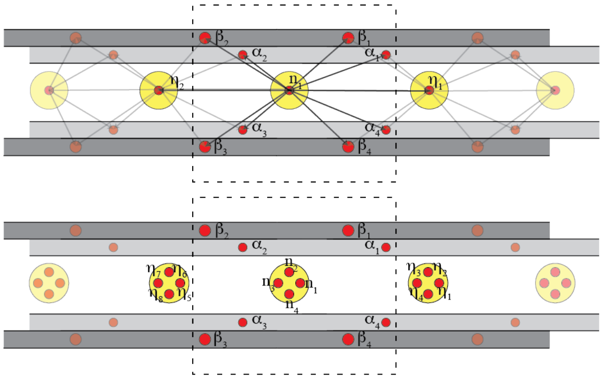

FIG. S7. **Force Vectors**. Labeling and depiction of force vectors for (top) single magnet per disk unit cell and (bottom) four magnets per disk unit cell. Grey lines represent the interactions between a unit cell, its boundary magnets, and its nearest neighbor.

nets, $f'(d)$ is the repulsive force's first derivative, $\mathbf{e}_{i,j}$ is the unit vector from magnet i to magnet j and $d_{i,j}$ is the distance between magnet i and magnet j. n is the index for disk magnets, η is the index of disk magnets in neighboring cells, α is the index for inner boundary magnets, β is the index for outer boundary magnets (See Fig. S7 for more details), a is the unit cell length, and $\otimes$ is the dyadic product. The smaller inner boundary magnets have a repulsive force $f(d)_s = A_s d^{\gamma_s}$ while the magnets in the outer boundaries and disks have repulsion force $f(d)_l = A_l d^{\gamma_l}$. Figure S7 depicts the magnetic force vectors of a single magnet per disk metamaterial and a four magnets per disk metamaterial. The force vectors depict the lowest order simulations which considers only the nearest neighbor interactions. For an N magnet per disk unit cell, labeling for each magnet in the unit cell is $n_i \in [1, N]$ while each magnet in the neighboring unit cell is $\eta_i \in [1, 2N]$ to consider the left and right nearest neighbor. Each unit cell consist of 4 inner boundary magnets $\alpha \in [1, 4]$ and 4 outer boundary magnets $\beta \in [1, 4]$.
